# Quantification and Comparison of Anti-Fibrotic Therapies by Polarized SRM and SHG-Based Morphometry in Rat UUO Model

**DOI:** 10.1371/journal.pone.0156734

**Published:** 2016-06-03

**Authors:** Hu Sheng Qian, Steve M. Weldon, Damian Matera, ChungWein Lee, Haichun Yang, Ryan M. Fryer, Agnes B. Fogo, Glenn A. Reinhart

**Affiliations:** 1 CardioMetabolic Disease Research, Boehringer Ingelheim Pharmaceutics, Inc, Ridgefield, Connecticut, United States of America; 2 Department of Pathology, Microbiology and Immunology, Vanderbilt University School of Medicine, Nashville, Tennessee, United States of America; Pennsylvania State Hershey College of Medicine, UNITED STATES

## Abstract

Renal interstitial fibrosis (IF) is an important pathologic manifestation of disease progression in a variety of chronic kidney diseases (CKD). However, the quantitative and reproducible analysis of IF remains a challenge, especially in experimental animal models of progressive IF. In this study, we compare traditional polarized Sirius Red morphometry (SRM) to novel Second Harmonic Generation (SHG)-based morphometry of unstained tissues for quantitative analysis of IF in the rat 5 day unilateral ureteral obstruction (UUO) model. To validate the specificity of SHG for detecting fibrillar collagen components in IF, co-localization studies for collagens type I, III, and IV were performed using IHC. In addition, we examined the correlation, dynamic range, sensitivity, and ability of polarized SRM and SHG-based morphometry to detect an anti-fibrotic effect of three different treatment regimens. Comparisons were made across three separate studies in which animals were treated with three mechanistically distinct pharmacologic agents: enalapril (ENA, 15, 30, 60 mg/kg), mycophenolate mofetil (MMF, 2, 20 mg/kg) or the connective tissue growth factor (CTGF) neutralizing antibody, EX75606 (1, 3, 10 mg/kg). Our results demonstrate a strong co-localization of the SHG signal with fibrillar collagens I and III but not non-fibrillar collagen IV. Quantitative IF, calculated as percent cortical area of fibrosis, demonstrated similar response profile for both polarized SRM and SHG-based morphometry. The two methodologies exhibited a strong correlation across all three pharmacology studies (r^2^ = 0.89–0.96). However, compared with polarized SRM, SHG-based morphometry delivered a greater dynamic range and absolute magnitude of reduction of IF after treatment. In summary, we demonstrate that SHG-based morphometry in unstained kidney tissues is comparable to polarized SRM for quantitation of fibrillar collagens, but with an enhanced sensitivity to detect treatment-induced reductions in IF. Thus, performing SHG-based morphometry on unstained kidney tissue is a reliable alternative to traditional polarized SRM for quantitative analysis of IF.

## Introduction

Renal interstitial fibrosis (IF) has been closely associated to loss of glomerular filtration rate (GFR) in chronic kidney disease (CKD). However, accurate, quantitative assessment of IF remains a challenge. Robust techniques to quantify IF in experimental models of CKD are important in the assessment of novel therapeutics that may impact progression of CKD [1].

One of the primary characteristics of IF is the accumulation of collagen and related molecules. Interstitial extracellular matrix (ECM) expansion is a hallmark of CKD, and increasing IF correlates with declining renal function and is often a predictor of CKD progression [1–2]. Non-fibrillar collagen type IV is a component of the ECM of both normal and diseased kidney tissue while fibrillar collagens type I and III are relatively disease specific. Thus, collagen types I and III are generally the primary collagen components used to quantify IF in fibrotic renal disease [1].

A variety of techniques have been used to measure IF. Common morphometric techniques used for assessment of IF are based on trichrome or Sirius Red staining and immunohistochemistry for type III collagen as an index of tissue collagen content [3–10]. Sirius Red morphometry (SRM) with polarized light is widely used for quantitative analysis of fibrillar collagen types I and III.

More recently, multiphoton microscopy based on two-photon excited fluorescence (TPEF) and second harmonic generation (SHG) has seen a surge in use in biomedical research [11–12]. Since SHG allows for the simultaneous visualization of tissue structure and fibrillar collagens in unstained tissue specimens, it offers some specific advantages compared to stain-based methods (e.g. trichome and SRM), such as elimination of stain-dependent variance and the ability to generate a 3D reconstruction of detailed IF from thick unstained samples [12–14]. SHG-based morphometry has been used to quantify fibrosis in skin, lung, liver and kidney tissue sections [15–22]. However, a rigorous comparison of polarized SRM and SHG-based morphometry in experimental models of IF has not been reported to date.

The rodent model of unilateral ureteral obstruction (UUO) model has been widely used to study mechanisms of IF and test novel anti-fibrotic therapies targeted for CKD [23–26]. In the present study, we compared polarized SRM and SHG-based morphometry for the measurement of IF using a rat UUO model in which the fibrotic disease process was ameliorated by pharmacologically targeting three distinct mechanisms. These treatments included the ACE inhibitor, enalapril (ENA), the immunosuppressant agent, mycophenolate mofetil (MMF), and the connective tissue growth factor (CTGF) neutralizing antibody, EX75606. Our results demonstrate that SHG-based morphometry is a sensitive method to quantify fibrillar specific collagen components in a model of experimental renal disease and may offer a wider dynamic range than polarized SRM to detect treatment-induced changes in IF.

## Materials and Methods

### Experimental design

Studies were performed in seven week old male Sprague Dawley rats, body weight averaging 220 grams. (Charles River Laboratories Inc, Stone Ridge, NY). Rats were housed under controlled temperature (22±1°C) and lighting (14:10h light-dark cycle) conditions with free access to normal rodent chow (Purina 5001, Ralston Purina, Richmond, IN) and water *ad libitum*. All experiments were conducted according to protocols approved by the Institutional Animal Care and Use Committee at Boehringer Ingelheim Pharmaceutics, Inc.

Animals were anesthetized with a mixture of 2% isoflurane with a flow rate of 2.5L oxygen/minute, and UUO was performed under aseptic conditions. Briefly, the left ureter and kidney were exposed through a midline abdominal incision; the ureter was isolated from the surrounding tissue and occluded using two 5–0 sterile sutures placed 2–3 mm apart and the upper suture placed parallel to the caudal boundary of the lower pole of the kidney. The ureter was cut between the 2 sutures to ensure permanent obstruction. Sham animals underwent similar surgical procedures whereby the left ureter was manipulated but without ligation. The abdominal incision was closed using 4–0 sterile suture followed by wound clips for skin closure. Rats regained consciousness quickly under post-operative supervision and were returned to fresh home cages for the duration of the study.

All drug treatments (n = 6/group and n = 6 for each vehicle control) began 24 hours prior to the surgical procedure and continued throughout the 5 day observation period, with specific vehicle controls. Drug-specific doses and treatment regimens were as follows: ENA (Spectrum Chemical, New Brunswick, NJ) was administered in the drinking water at doses of 15, 30, or 60 mg/kg/day; MMF (Sandoz Inc, Princeton, NJ) was administered via oral gavage once a day at doses of 2 or 20 mg/kg/day (vehicle = 0.5% methylcellulose / 0.015% Tween 80; Spectrum Chemical, New Brunswick, NJ); EX75606 (a human monoclonal antibody against CTGF generated at Boehringer Ingelheim Pharmaceutics, Inc. by using FibroGen’s patent of FG3019) was administered every other day as a single i.p. injection at doses of 1, 3, or 10 mg/kg (vehicle = citrate buffer).

At the end of the study, animals were sacrificed by exsanguination via cardiac puncture under deep general anesthesia with 3.5% isoflurane; kidneys and ureters were exposed through an abdominal incision. Both kidneys were removed and immediately fixed by immersion in 10% phosphate-buffered formalin for 48 hours; formalin-fixed kidneys were then rinsed in phosphate buffer, dehydrated via a graded series of ethanol and xylene, embedded in paraffin, and sagittally sectioned at 4μm. The right kidney served as the control to the left obstructed kidney.

Immunohistochemistry (IHC) and SHG-based morphometry was done at Boehringer Ingelheim, while SR staining and polarized SRM analysis was performed at Vanderbilt University.

### Immunohistochemistry of Collagen I, III, and IV

Kidney sections were air dried overnight at 37°C, dewaxed and rehydrated in graded ethanol to phosphate buffered saline (PBS). Endogenous peroxidase activity was blocked by immersion in 3.0% hydrogen peroxide and 0.1% sodium azide in PBS for 10 min at ambient temperature. Sections were washed three times in PBS for 5 min, incubated for 20 min with normal goat serum diluted 1:10 with 0.1 mol/L PBS, pH 7.4, then incubated with the primary antibody against collagen I (ab34710, Abcam, Cambridge, MA) at a dilution of 1:250, collagen III (ab34710, Abcam, Cambridge, MA) at a dilution of 1:600, or collagen IV (ab34710, Abcam, Cambridge, MA) at a dilution of 1:250 for overnight (18 h) at 4°C. All sections were subsequently incubated with the respective secondary antibodies at room temperature for 1 h. Peroxidase-labeled polymer and substrate–chromogen were then employed to visualize staining. The primary antibody was replaced with PBS as negative control

### Sirius Red staining and morphometric analysis

Sections were deparaffinized using xylene and absolute ethanol, rehydrated with tap water, stained with 0.1% Sirius Red F3BA solution (Sigma-Aldrich, St. Louis, MO) in saturated aqueous picric acid (Sigma-Aldrich, St. Louis, MO) overnight at room temperature, washed in 0.01 N hydrochloric acid for 2 min, dehydrated in 3 changes of 100% ethanol, cleared in xylene and mounted on cover slips in a resinous medium.

All image acquisition and morphometric analyses were performed under blinded conditions. Sirius Red staining was examined with an Olympus BX-41 microscope (Melville, NY, USA). To optimize polarized light conditions, the analyzer (upper polarizing filter) was fixed, so that its transmission axis was aligned at 45° to the fixed axis of the quarter plate above it. The circular polarizer (lower polarizing filter) was rotated and aligned at an angle of 90° to the analyzer, so that the field of view was dark. Optimal polarization with dark field was recalibrated with a standard test slide before capture of each series of new sections. Under objective 20x 0.40NA (CFI Plan, WD = 1.2mm, Nikon, Japan), an Olympus DP-72 CCD camera (Melville, NY, USA) was used to capture images. Morphometry was performed using AxioVision 4.7.2.0 (Carl Zeiss Microscopy, Germany). During image acquisition, the capsule and medulla were excluded from the visual field and an average of 20 images (2584 X 1936 pixel spatial resolution with the pixel size of 0.2 μm x 0.2μm) were collected for each kidney. For quantitative analysis of IF, the background intensity threshold was set automatically by using the hue (H 5–148), saturation (S 4–255), and brightness (B 70–255). After manually outlined to define the region of interest (ROI), perivascular structures were excluded from images, so fibrillar collagens detected were not of vascular origin. The values of cortical IF for each animal were calculated by the number of pixels showing intensity above the background threshold relative to the total number of pixels within the ROI. The average percent area of cortic fibrosis was generated from 20 individual images per animal. Quantitative IF for each group was expressed as the mean value ± SEM.

### TPFE-SHG imaging and morphometric analysis

TPFE and SHG imaging were performed with cover slips on unstained, deparaffinized, 4 μm kidney sections with a Zeiss LSM 7MP upright laser scanning system (Carl Zeiss Microscopy, Germany), coupled to a mode-locked femtosecond Ti: sapphire laser (80 MHz), tunable from 680 to 1020 nm (Chameleon Vision II Laser System, Coherent, Santa Clara, CA). Excitation wavelength of 860 nm was used with average power of ~10–12 mW at the specimen using a long working distance water-dipping objective 10×0.45NA (C-Apochromat, WD = 1.8mm, M27, Zeiss Microscopy, Germany).SHG images were taken using 2P-blocked bandpass emission filter (HQ515/20m-2P, Chroma Technology Corp, Bellows Falls, VT) and TPEF images were taken using bandpass filter (BP565-610, Zeiss Microscopy, Germany). All SHG and TPEF images were acquired simultaneously by non-descanned detector (NDD) in the forward direction. To remove background, the 32-bit image was auto-thresholded to mean intensity by using the Zeiss auto-exposure algorithm (Laser power = 46%, NDD master gain = 775, digital offset = -213, and digital gain = 1.31). Laser intensity and gain were calibrated with a standard reference section prior to each experiment. An average of 28 images (1024 X 1024 pixel spatial resolution with the pixel size of 1.06 μm x1.06μm) was acquired per kidney and all morphometric were assessments performed in a blinded manner identical to that used for SRM.

To compare measurements between SRM and SHG-based morphometry, the dynamic range of response for UUO-induced IF in vehicle group and response for anti-fibrotic regimens in each treatment group was calculated as the ratio of the mean value between obstructed kidney and contralateral kidney.

### Statistical Analysis

All values are expressed as the mean ± SEM. One-way ANOVA with Dunnett’s multiple comparisons test was used to compare the treatment groups with the respective vehicle control. The correlations for the results of percent cortical fibrotic area measured by polarized SRM and SHG-based morphometry for all 3 intervention studies were evaluated by nonlinear regression analysis. *P*< 0.05 was considered statistically significant. Statistical analyses were performed using GraphPad Prism 6 statistical software (GraphPad Software, La Jolla, CA)

## Results

**Fig 1:**
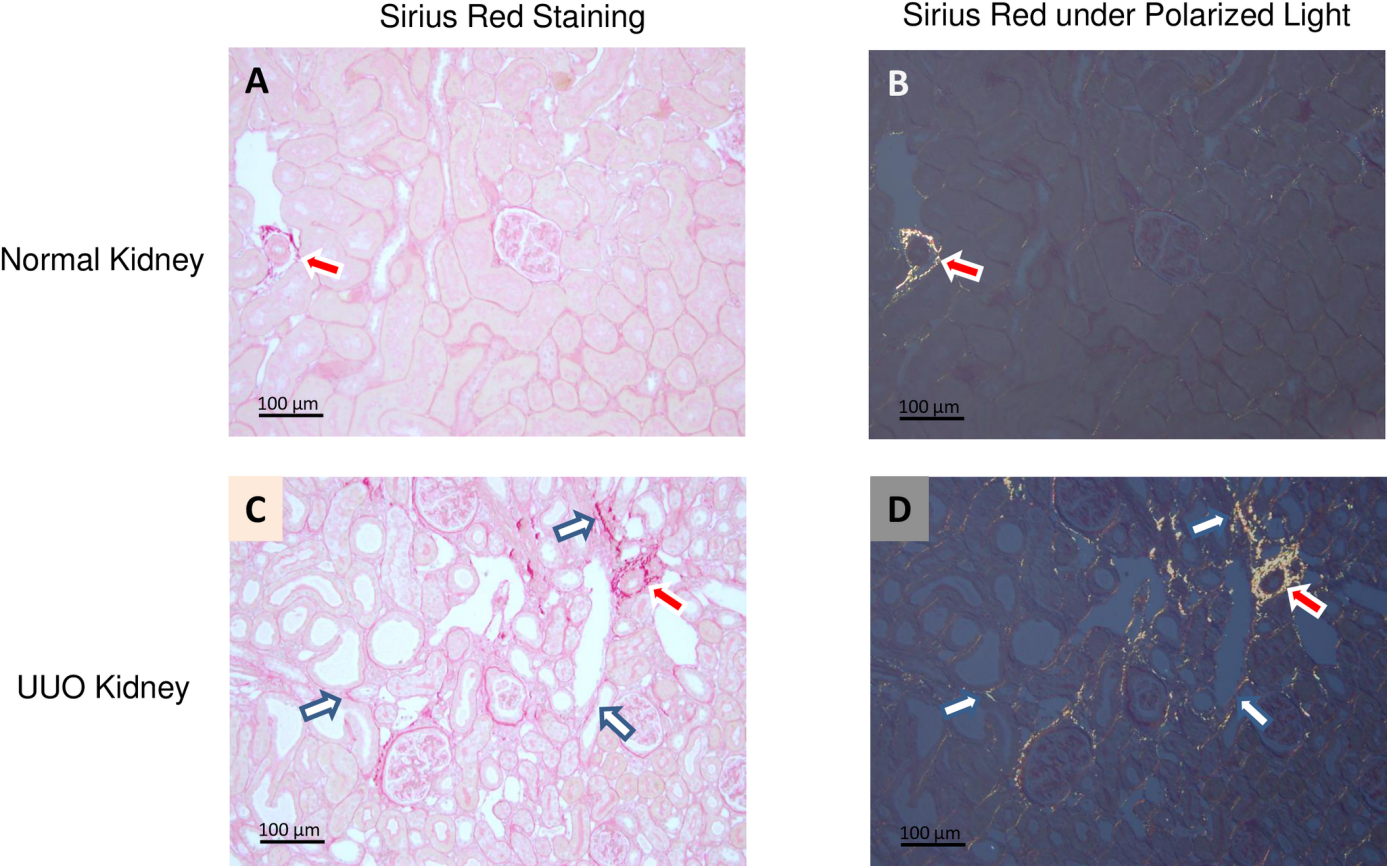
Representative Sirius Red staining of renal IF: Sirius Red staining was visualized by either transmitted light microcopy or under polarized light microscopy in normal rat kidney (Fig 1A and Fig 1B) and the 5 day UUO kidney (Fig 1C and Fig 1D). Under polarized light, fibrillar collagen appears as yellow structures; both adventitial vasculature (red arrow) and tubulointerstitum (white arrow) are easily identified. The scare bar in each image is 100μm, and the object is oriented at ±45° between crossed polar, and either the polarizer or the analyzer is rotated through an equal angle (Fig 1B and Fig 1D).

The Sirius Red molecule intercalates into the tertiary groove of collagen molecules, including collagen types I, III, and IV, and imparts a pink stain to stained kidney sections under white light (Fig 1A for normal kidney and Fig 1C for UUO kidney). When the same sections are observed under polarized light, fibrillar collagen types I and III are strongly birefringent and visualized as yellow structures (Fig 1B for normal kidney and Fig 1D for UUO kidney). In the normal kidney, only trace amounts of collagen are detected between tubules (Fig 1B). In contrast, significant interstitial collagen deposition is visible in UUO kidney sections (Fig 1D, white arrow). As expected, perivascular structures and adventitia exhibit a substantial collagen component in both normal and UUO kidneys (Fig 1B and 1D, red arrow). These structures are clearly visible and were excluded from all morphometric analyses.

**Fig 2:**
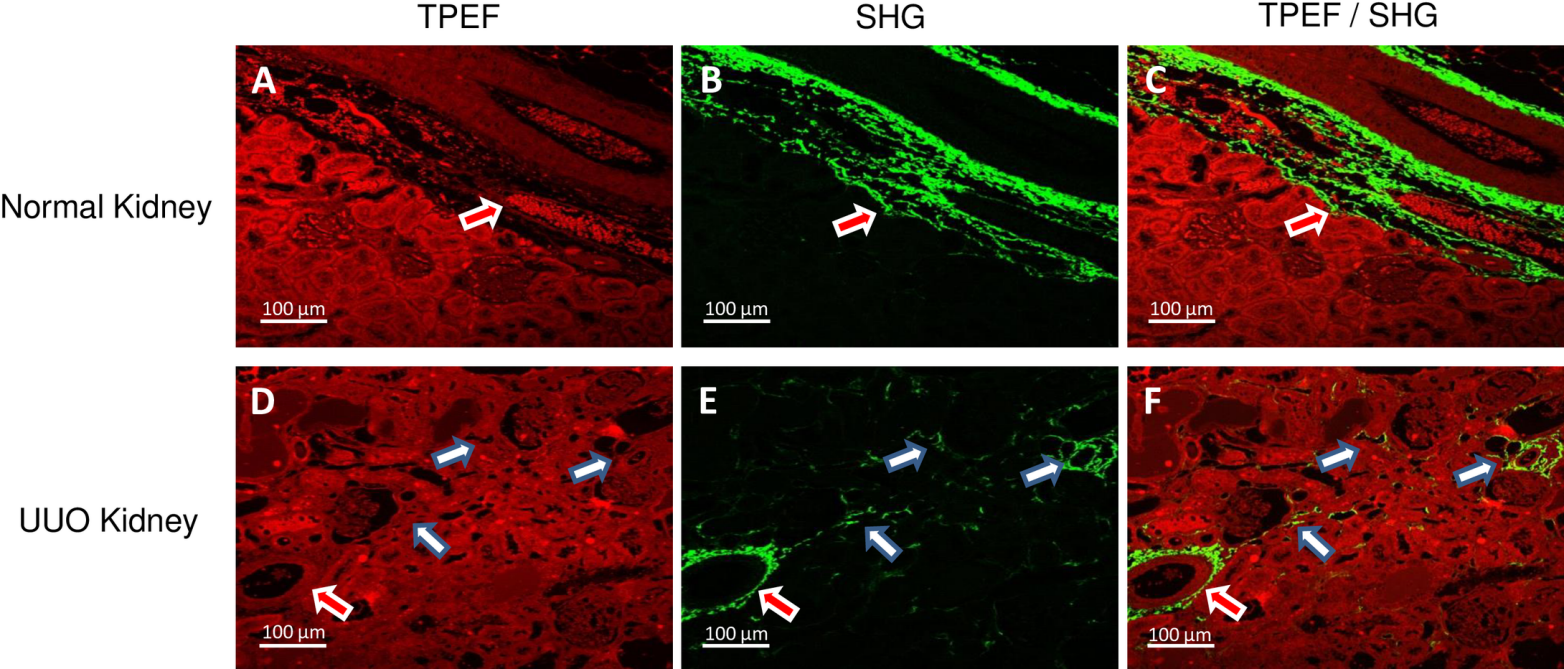
Representative TPEF / SHG imaging of renal IF: By using unstained, de-parrafinized kidney sections, TPEF imaging shows autofluorescence with discernible kidney structure (Fig 2A and Fig 2D with red color), while the SHG signal specifically visualizes fibrillar collagens in both adventitia of blood vessels (red arrow) and tubulointerstitium (white arrow) (Fig 2B and Fig 2E with green color). Simultaneous TPEF and SHG imaging clearly reveal fibrillar collagen distribution (Fig 2C and Fig 2F). Note that all images were taken in the forward direction, and SHG images were taken using 2P-blocked bandpass emission filter (HQ515/20m-2P, Chroma Technology Corp, Bellows Falls, VT) and TPEF images were taken using bandpass filter (BP565-610, Zeiss Microscopy, Germany). The scare bar in each image is 100μm.

For TPEF-SHG imaging, unstained, deparaffinized kidney sections were used for the simultaneous visualization of structure and fibrillar collagen. TPEF imaging shows the typical autofluorescence and discernible kidney structure (red color; Fig 2A for normal kidney; Fig 2D for UUO), but SHG signal (green) specifically visualized only fibrillar collagens in both the adventitia of arteries (red arrow) and tubulointerstitium (white arrow; Fig 2B and 2E). Co-localized TPEF and SHG imaging clearly revealed the distribution of fibrillar collagens in both normal and UUO kidney sections (Fig 2C and 2F).

**Fig 3:**
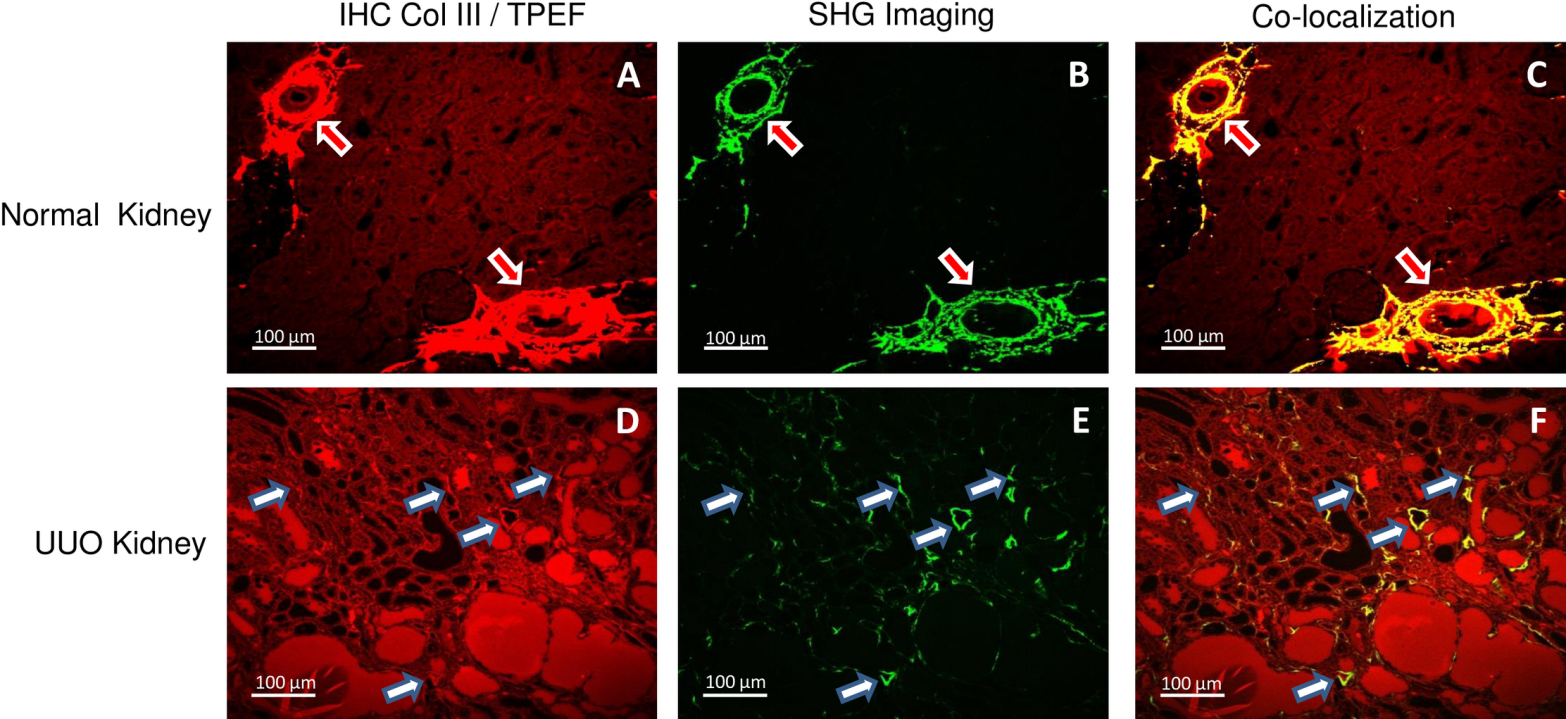
Co-locolization of SHG signal with IHC for fibrillar collagen III: In normal kidney, only adventitia of artery and large vein demonstrated positive IHC signal for collagen III (Fig 3A), however, for the UUO kidney section, many thin and scattered signals were captured in the interstitial area (Fig 3D). A similar expression pattern was observed using SHG (B and E), and confirmed by co-localized of both the SHG and IHC staining (Fig 3C and Fig 3F). Note that all images were taken in the forward direction, and SHG images were taken used 2P-blocked bandpass emission filter (HQ515/20m-2P, Chroma Technology Corp, Bellows Falls, VT) and TPEF images were taken used bandpass filter (BP565-610, Zeiss Microscopy, Germany). The scare bar in each image is 100μm.

To validate the selectivity of SHG-based morphometry for fibrillar collagens, we performed immunohistochemistry for collagen types I, III, IV to test for co-localization with distribution of the SHG signal. As shown in Fig 3, the SHG signal strongly co-localized with fibrillar collagen III in both adventitia of blood vessels and the tubulointerstitium as identified by IHC. Collagen I also co-localized with SHG and displayed a similar distribution to collagen III, whereas no significant co-localization was observed for non-fibrillar collagen IV (data not shown).

In order to assess the reproducibility of SHG signal analysis, repeated image acquisition and morphometric analysis were performed on the same kidney sections and locations at different days (1, 3, and 7 days apart). The IF signals after repeated measurements were nearly identical, suggesting no appreciable photo bleaching after multiple, time-dependent rounds of image acquisition and analysis. (S1 Table).

**Fig 4:**
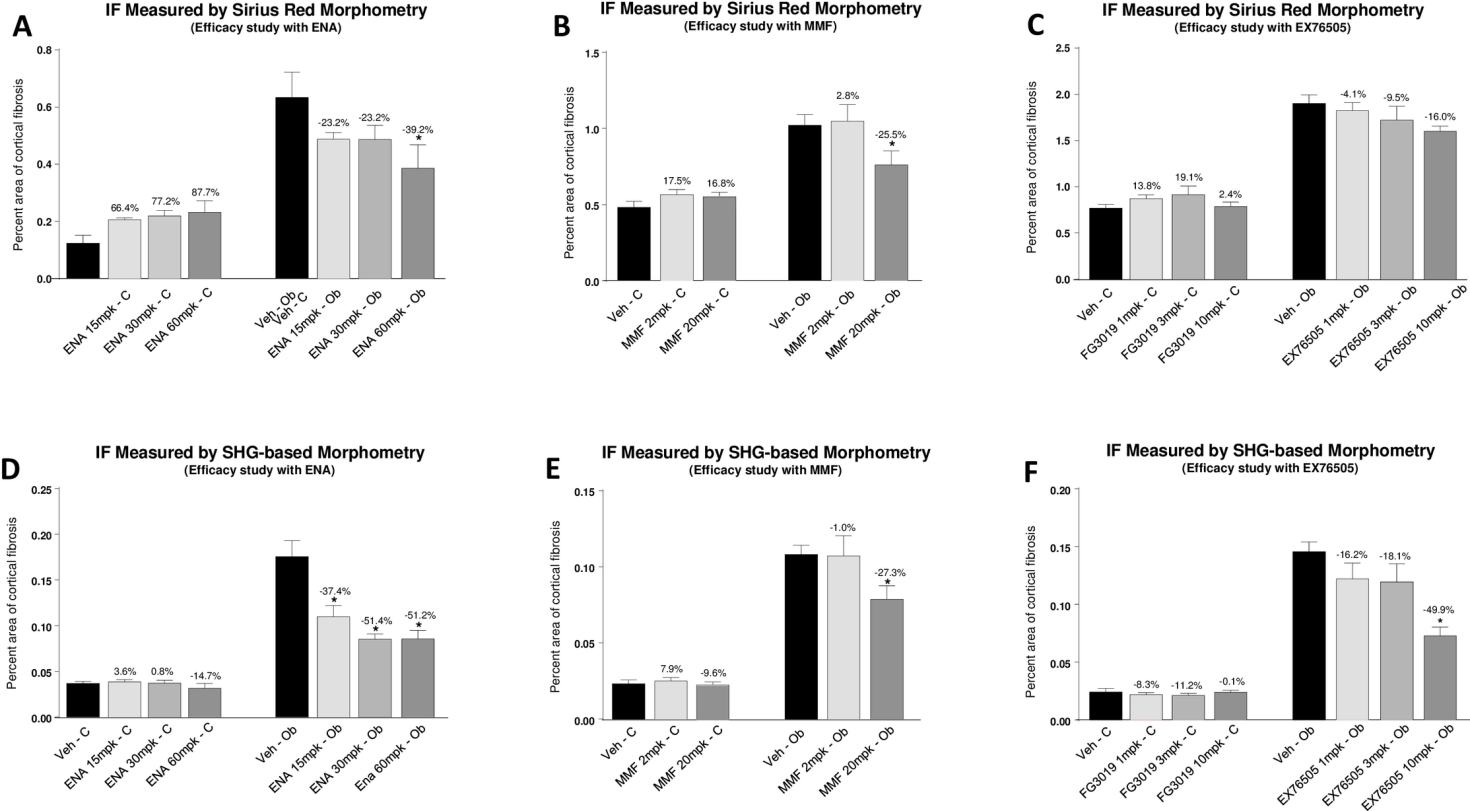
Comparison of response of therapeutic anti-fibrotic effects evaluated by SRM and SHG-based mephometry: Rats with UUO were treated with ACE inhibitor (enalapril), the immunosuppresant agent (mycophenolate mofetil), or CTGF neutralizing antibody. Treatment-induced reduction in IF was evaluated either by polarized SRM (A, B, and C) or by SHG-based morphometry (D, E, and F).

**Fig 5:**
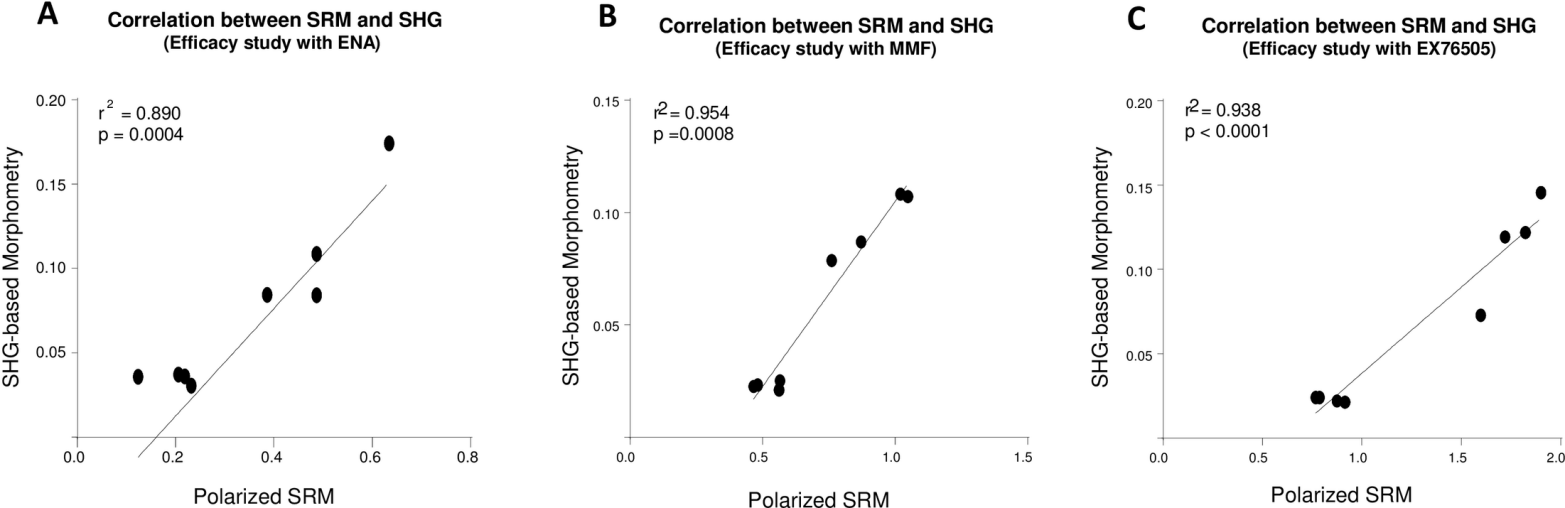
Correlation between polarized SRM analysis and SHG-based morphometry: In all three UUO treatments, polarized SRM and SHG-based morphometry correlated strongly.

In studies performed to assess the correlation between polarized SRM and SHG-based morphometry to quantify IF in the rat UUO model, the results of quantitative IF measured by polarized SRM and SHG-based morphometry demonstrated similar trends of response profile (Fig 4). Correlation between the methods was confirmed within each study independent of dose of the treatment drug (Fig 5, ENA: r^2^ = 0.890, p = 0.0004; MMF: r^2^ = 0.954, p = 0.0008; EX76505: r^2^ = 0.938, p<0.0001). However, the methodologies differed with respect to the overall magnitude of anti-fibrotic effect based on quantitative IF measurements and ability to detect statistically-significant changes in IF after treatment. In general, the therapeutic window between vehicle group and each treatment group measured by polarized SRM was smaller than that measured by SHG-based morphometry. In UUO studies performed with ENA at 15, 30, and 60 mg/kg, ENA reduced IF 23.2–39.2% based on SRM, vs 37.4–51.4% reduction based on SHG. While both methodologies produced a similar magnitude reduction in IF after treatment with 20 mg/kg MMF (25.5% vs. 27.3% reduction with SRM and SHG, respectively); the CTGF inhibitor, EX76505, at 1, 3, and 10 mg/kg, produced a 4.1–16.0% reduction based on SMR vs 16.2–49.9% based on SHG. Further, a statistically significant effect at 10 mg/kg was only detected by SHG. Lastly, for the contralateral kidney, compared with animals treated with vehicle, changes in quantitative IF in each study measured by SHG-based morphometry was consistently smaller and less variable than the value obtained by SRM (from IF increase of 7.9% to reduction of 14.7% based on SHG, vs from an increase of 87.7% IF to reduction of 3.2% based on SRM).

To compare the sensitivity of measurement between SRM and SHG-based morphometry, the dynamic range of quantitative IF between the obstructed kidney and contralateral kidney was calculated. Our results demonstrate that both the response of UUO-induced IF in the vehicle group, and the response for anti-fibrotic regimens in treatment groups, exhibited similar trends. However, the dynamic range of quantitative IF evaluated by polarized SRM was smaller (Table 1, 1.4 to 5.1 fold, average 2.3 fold) than SHG-based morphometry across all 3 studies (Table 1, 2.3 to 6.0 fold, average 4.1 fold).

**Table 1:** Comparison of the dynamic range of response between polarized SRM and SHG-based morphometry.

	Efficacy study with ENA				Efficacy study with MMF			Efficacy study with EX76505				Average (fold)
	Veh	15 mg/kg	30 mg/kg	60 mg/kg	Veh	2 mg/kg	20 mg/kg	Veh	1 mg/kg	3 mg/kg	10 mg/kg	
**SRM**	5.1	2.4	2.2	1.7	2.1	1.9	1.4	2.5	2.1	1.9	2.0	2.3
**SHG**	4.8	2.9	2.3	2.7	4.6	4.3	3.7	6.0	5.5	5.6	3.0	4.1

Veh: vehicle; ENA: enalapril; MMF: mycophenolate mofetil; EX76505: CTGF neutralized antibody.

## Discussion

In the present study, we compare and contrast two methods, polarized SRM and SHG-based morphometry, to measure IF in an experimental model of renal disease, rat UUO. We demonstrate that SHG-based morphometry is selectively detecting fibrillar collagen type I and III. However, while strong correlations were observed between the two imaging techniques across three studies, we demonstrate that changes in IF due to pharmacological modulation could be more sensitively detected with SHG-based morphometry, at least in part, due to a wider dynamic range for detection of a therapeutic effect.

Sirius Red is a histochemical stain that has been used for over a half century [27]. Quantitative analysis of fibrillar collagens using polarized SRM has been widely used for both animal models and human biopsy samples [4–7, 28]. In contrast, the first SHG imaging of fibrillar collagen was reported in the 1980’s [29], and SHG-based morphometry was introduced only a decade ago [14]. Compared with the traditional morphometric methods such as SRM, trichrome, and immunohistochemistry of collagen I / III, SHG-based morphometry may offer several advantages for assessing IF in the preclinical setting. Using the rat UUO model, in the present study, we confirmed the results of previous studies [16, 20, 30] that demonstrated SHG specifically identified fibrillar collagen, typically collagen type I and III, while non-fibrillar collagen type IV was not detected, suggesting the potential for significantly reduced background, and increased signal-to-noise ratio (SNR), which may have contributed to the wider dynamic range observed in the rat UUO studies. Moreover, SHG-based morphometry can be applied to *in vivo* and *ex vivo* studies, and performed in unstained tissue from fresh, frozen, or fixed tissues, providing a simplified work flow and reducing potential variations caused by the staining process [16–18, 31–33]. Finally, due to the characteristics of deeper penetration and greater resolution, SHG-based morphometry can be used to generate 3D reconstruction of the fibrillar collagen to quantify details of IF based on thicker tissue sample [16, 34–36] and thus, can be used to quantify IF in whole human kidney biopsy samples [17,36].

In the present study, we demonstrated in the rat UUO model of IF, that SHG-based morphometry has good reproducibility for the measurement of IF. As confirmed by the co-localization of immunohistochemistry specific staining of collagen I and III, we validated the specificity of SHG for detecting fibrillar collagen components in IF. Based on the distribution of fibrillar collagen observed by polarized SRM and SHG-based morphometry techniques in both UUO and contralateral kidneys was near identical, we demonstrated SHG-based morphometry in unstained kidney tissues is comparable to polarized SRM for quantitation of fibrillar collagens.

The rat UUO model is an experimental model of rapid and progressive IF commonly used for investigating novel mechanistic pathways involved in IF and validating novel therapeutic interventions. Thus, technical improvements in the accurate detection of IF, as we demonstrate with SHG-based morphometry, may enable more definitive assessment in the effectiveness of new anti-fibrotic therapies to modulate the disease process. Indeed, while the ACE inhibitor, enalapril, and the immunosuppressant agent, MMF, have been shown to attenuate IF following UUO in rats [37,38], only one study using mouse UUO has been reported to evaluate an anti-fibrotic effect of the CTGF neutralizing antibody (FG3019). However, in the latter study a significant reduction of renal collagen deposition was measured only by assessment of the hydroxyproline: proline (Hyp:Pro) ratio [39]. Moreover, all of these studies employed a 14 day UUO model, and for the measurement of IF, none reported quantitative morphometric analysis.

In the present study, quantitative IF measured by polarized SRM and SHG-based morphometry showed similar response profiles and the two methodologies exhibited a strong correlation across three pharmacology studies. In addition, the anti-fibrotic effect measured by both techniques also demonstrated a dose-responsive reduction in IF in all three treatment groups. Thus, our results indicated both polarized SRM and SHG-based morphometry are highly sensitive in detecting small changes of fibrillar collagens in the rat UUO model, and confirmed the ability of SHG-based morphometry to provide accurate analysis of IF in a model of kidney fibrosis.

The findings that SHG-based morphometry had a larger dynamic range and a corresponding greater treatment-induced reduction in IF may be due to the fact that fibrillar collagen is highly noncentrosymmetric and possesses a very high nonlinear susceptibility. Under SHG microscopy, fibrillar collagen produces bright and robust signals. Consequently, very thin fibrils of collagen are highly visible as a source of light against a dark background by SHG. Thus, compared with traditional morphometric techniques, SHG provided a higher signal-to-noise ratio, which is a key factor for the sensitivity and accuracy of imaging analysis [40]. A lower signal-to-noise ratio in SRM may also be due to some degree of non-specific signals that, in the present study, is supported by higher level of fibrillar collagen in the contralateral kidney detected by polarized SRM.

Although SHG-based morphometry offers some special advantages for quantitative imaging analysis of renal IF compared with polarized SRM, it also has disadvantages: First, it is not likely to be considered as a routine technique due to appreciable complexity and high cost [41]. Second, because SHG signal intensity technically is highly dependent on the laser power, laser power has to be closely monitored to avoid potential variability for each study [20]. Lastly, compared with the current digital pathology technology, such as the Aperio whole slide scanner and the Vectra automated multispectral imaging system, which can process up to 400 slides for quantitative analysis of SRM and IHC [42], SHG-based morphometry has the same limitation of low throughput as does polarized SRM. This may limit the utility of SHG-based morphometry in the drug discovery environment in which a large number of samples are often assessed. However, future integration of automation with SHG-based morphometry has the potential to significantly increase its throughput.

However, one limitation of the present study is that because SRM and SHG-based morphometry were independently optimized and performed at two different labs, the resolution and pixel size of the images used for each modality were different (see Methods). While these differences have the potential to produce slightly different quantitative values for IF calculations, it is clear that the strong degree of correlation of obtained across the three pharmacology studies remains robust.

In conclusion, the present study demonstrates that SHG-based morphometry in unstained kidney tissues is comparable to polarized SRM for quantitation of fibrillar collagens in an experimental model of progressive IF, and may offer increased sensitivity for detecting treatment-induced reductions in IF. Moreover, we demonstrate for the first time the antifibrotic potential of the CTGF antibody, EX75606, to attenuate fibrosis in rat UUO based on quantitative IF, suggesting that this pathway could be targeted for therapeutic treatment of renal fibrotic diseases.

## Supporting Information

S1 Table Reproducibility of SHG-based morphometry on the same kidney sections. The values of cortical IF were calculated by the number of pixels showing intensity above the background threshold relative to the total number of pixels within the ROI.
(TIF)

## References

[1] Farris AB, Chan S, Climenhaga J, Adam B, Bellamy CO, Serón D, et al Banff Fibrosis Study: Multicenter visual sssessment and computerized analysis of interstitial fibrosis in kidney biopsies. Am J Transplant 2014; 14: 897–907 10.1111/ajt.12641 24712330

[2] Liu Y. Renal fibrosis: New insights into the pathogenesis and therapeutics. Kidney Int 2006; 69: 213–217 16408108 10.1038/sj.ki.5000054

[3] Boor P, Ostendorf T, Floege J. Renal fibrosis: Novel insights into mechanisms and therapeutic targets. Nat Rev Nephrol 2010; 6:643–656. 10.1038/nrneph.2010.120 20838416

[4] Farris AB, Adams CD, Brousaides N, Pelle PAD, Collins AB, Moradi E, et al Morphometric and visual evaluation of fibrosis in renal biopsies. J Am Soc Nephrol 2011; 22: 176–186 10.1681/ASN.2009091005 21115619 PMC3014046

[5] Junqueira LC, Bignolas G, Brentani RR. Picrosirius staining plus polarization microscopy, a specific method for collagen detection in tissue sections. Histochem J 1979; 11: 447–455 91593 10.1007/BF01002772

[6] Grimm PC, Nickerson P, Gough J, McKenna R, Stern E, Jeffery J, et al Computerized image analysis of sirius red-stained renal allograft biopsies as a surrogate marker to predict long-term allograft function. J Am Soc Nephrol 2003; 14: 1662–1668 12761269 10.1097/01.asn.0000066143.02832.5e

[7] Sund S, Grimm P, Reisaeter AV, Hovig T. Computerized image analysis vs semiquantitative scoring in evaluation of kidney allograft fibrosis and prognosis. Nephrol Dial Transplant 2004;19: 2838–2845 15385637 10.1093/ndt/gfh490

[8] Feldman DL, Mogelesky TC, Chou M, Jeng AY. Enhanced expression of renal endothelin-converting enzyme-1 and endothelin-A-receptor mRNA in rats with interstitial fibrosis following ureter ligation. J Cardiovasc Pharmacol 2000;36: S255–S259 11078391 10.1097/00005344-200036051-00075

[9] Satoh M, Kashihara N, Yamasaki Y, Maruyama K, Okamoto K, Maeshima Y, et al Renal interstitial fibrosis is reduced in angiotensin II type 1a receptor-deficient mice. J Am Soc Nephrol 2001;12: 317–325 11158221 10.1681/ASN.V122317

[10] Fogo AB, Alpers CE. Navigating the challengers of fibrosis assessment: land in sight? J Am Soc Nephrol 2011;22:11–13 10.1681/ASN.2010111132 21127139

[11] Zipfel WR, Williams RM, Webb WW. Nonlinear magic: multiphoton microscopy in the biosciences. Nat Biotechnol 2003;21:1369–1377. 14595365 10.1038/nbt899

[12] Chen G, Chen J, Zhuo S, Xiong S, Zeng H, Jiang X, et al Nonlinear spectral imaging of human hypertrophic scar based on two-photon excited fluorescence and second-harmonic generation. Br J Dermatol 2009;161:48–55. 10.1111/j.1365-2133.2009.09094.x 19309369

[13] Campagnola PJ, Clark HA, Mohler WA, Lewis A, Loew LM. Second-harmonic imaging microscopy of living cells. J Biomed Opt 2001;6:277–286. 11516317 10.1117/1.1383294

[14] Campagnola PJ, Loew LM. Second-harmonic imaging microscopy for visualizing biomolecular arrays in cells, tissues and organisms. Nat Biotechnol 2003;21:1356–1360. 14595363 10.1038/nbt894

[15] Zoumi A, Yeh A, Tromberg BJ. Imaging cells and extracellular matrix in vivo by using second-harmonic generation and twophoton excited fluorescence. Proc Natl Acad Sci USA 2002; 99:11014–19. 12177437 10.1073/pnas.172368799 PMC123202

[16] Gailhouste L, Le Grand Y, Odin C, Guyader D, Turlin B, Ezan F, et al Fibrillar collagen scoring by second harmonic microscopy: a new tool in the assessment of liver fibrosis. J Hepatol 2010;52:398–406 10.1016/j.jhep.2009.12.009 20149472

[17] Strupler M, Pena AM, Hernest M, Tharaux PL, Martin JL, Beaurepaire E, et al Second harmonic imaging and scoring of collagen in fibrotic tissues. Opt Express 2007;15:4054–65. 19532649 10.1364/oe.15.004054

[18] Strupler M, Hernest M, Fligny C, Martin JL, Tharaux PL, Schanne-Klein MC. Second harmonic microscopy to quantify renal interstitial fibrosis and arterial remodeling. J Biomed Opt 2008;13:054041 10.1117/1.2981830 19021421

[19] Zipfel WR, Williams RM, Christie R, Nikitin AY, Hyman BT, Webb WW. Live tissue intrinsic emission microscopy using multiphoton-excited native fluorescence and second harmonic generation. Proc Natl Acad Sci USA 2003;100:7075–7080. 12756303 10.1073/pnas.0832308100 PMC165832

[20] Strupler M, Pena AM, Hernest M, Tharaux PL, Martin JL, Beaurepaire E, et al Second harmonic imaging and scoring of collagen in fibrotic tissues. Opt Express 2007;15:4054–65. 19532649 10.1364/oe.15.004054

[21] Sun W, Chang S, Tai DC, Tan N, Xiao G, Tang H, et al Nonlinear optical microscopy: use of second harmonic generation and two-photon microscopy for automated quantitative liver fibrosis studies. J Biomed Opt 2008;13:064010 10.1117/1.3041159 19123657

[22] Deka G, Wu WW, Kao FJ. In vivo wound healing diagnosis with second harmonic and fluorescence lifetime imaging. J Biomed Opt 2013;18:061222 23264966

[23] Chevalier RL, Forbes MS, Thornhill BA. Ureteral obstruction as a model of renal interstitial fibrosis and obstructive nephropathy. Kidney Int 2009;75:1145–52 10.1038/ki.2009.86 19340094

[24] Yang HC, Zuo Y, Fogo AB. Model of Chronic kidney disease. Drug Discov Today Dis Models 2010;7:13–19. 21286234 10.1016/j.ddmod.2010.08.002 PMC3030258

[25] Forbes MS, Thornhill BA, Chevalier RL. Proximal tubular injury and rapid formation of atubular glomeruli in mice with unilateral ureteral obstruction: a new look at an old model. Am J Physiol Renal Physiol 2011;301:F110–7 10.1152/ajprenal.00022.2011 21429968 PMC3129891

[26] Bascands JL, Schanstra JP. Obstructive nephropathy: insights from genetically engineered animals. Kidney Int 2005;68:925–37 16105023 10.1111/j.1523-1755.2005.00486.x PMC1885837

[27] Sweat F, Puchtler H, Rosenthal SI. Sirius red F3BA as stain for connect tissue. Arch Pathol 1964;78: 69–72SWEA 14150734

[28] Street JM, Souza AC, Alvarez-Prats A, Horino T, Hu X, Yuen PS, et al Automated quantification of renal fibrosis with sirius red and polarization contrast microscopy. Physiol Rep 2014;2:e12088 10.14814/phy2.12088 25052492 PMC4187565

[29] Freund I, Deutsch M, Sprecher A. Connective tissue polarity. Optical second-harmonic microscopy, crossed-beam summation, and small-angle scattering in rat-tail tendon. Biophys J 1986;50:693–712 3779007 10.1016/S0006-3495(86)83510-X PMC1329848

[30] Pirhonen J, Arola J, Sadevirta S, Luukkonen P, Karppinen SK, Pihlajaniemi T, et al Continuous grading of early fibrosis in NAFLD using label-free imaging: A proof-of-concept study. PLoS One 2016 1 25;11(1):e0147804 10.1371/journal.pone.0147804 26808140 PMC4726624

[31] Schenke-Layland K, Riemann I, Damour O, Stock UA, König K. Two-photon microscopes and in vivo multiphoton tomographs—powerful diagnostic tools for tissue engineering and drug delivery. Adv Drug Deliv Rev 2006;58:878–96 17011064 10.1016/j.addr.2006.07.004

[32] Schenke-Layland K. Non-invasive multiphoton imaging of extracellular matrix structures. J Biophotonics 2008;1:451–62 10.1002/jbio.200810045 19343671 PMC4350994

[33] Strachan CJ, Windbergs M, Offerhaus HL. Pharmaceutical applications of non-linear imaging. Int J Pharm 2011;417:163–72 10.1016/j.ijpharm.2010.12.017 21182913

[34] Cox G, Kable E, Jones A, Fraser I, Manconi F, Gorrell MD. 3-Dimensional imaging of collagen using second harmonic generation. J Struct Biol 2003;141:53–62. 12576020 10.1016/s1047-8477(02)00576-2

[35] Pena AM, Fabre A, Debarre D, Marchal-Somme J, Crestani B, Martin JL, et al Three-dimensional investigation and scoring of extracellular matrix remodeling during lung fibrosis using multiphoton microscopy. Microsc Res Tech 2007;70:162–170. 17177275 10.1002/jemt.20400

[36] Torres R, Velazquez H, Chang JJ, Levene MJ, Moeckel G, Desir GV and Safirstein R. Three-dimensional morphology by multiphoton Microscopy with clearing in a model of cisplatin-induced CKD. J Am Soc Nephrol 2015;27:26303068 10.1681/ASN.2015010079 PMC4814184

[37] Chen CO, Park MH, Forbes MS, Thornhill BA, Kiley SC, Yoo KH, et al Angiotensin-converting enzyme inhibition aggravates renal interstitial injury resulting from partial unilateral ureteral obstruction in the neonatal rat. Am J Physiol Renal Physiol 2007;292:F946–955 17107943 10.1152/ajprenal.00287.2006

[38] Yuan XP, He XS, Wang X, Liu LS, Fu Q. Triptolide attenuates renal interstitial fibrosis in rats with unilateral ureteral obstruction. Nephrology 2011;16:200–210 10.1111/j.1440-1797.2010.01359.x 21272133

[39] Wang QJ, Usinger W, Nichols B, Gray J, Xu L, Seeley JW, et al Cooperative interaction of CTGF and TGF-β in animal models of fibrotic disease. Fibrogenesis & Tissue Repair 2011;4;4 21284856 10.1186/1755-1536-4-4 PMC3042008

[40] Martin TP, Norris G, McConnell G, Currie S. A novel approach for assessing cardiac fibrosis using label-free second harmonic generation. Int J Cardiovasc Imaging 2013; 29:1733–1740 10.1007/s10554-013-0270-2 23921804

[41] Bedossa Pierre. Harmony in liver fibrosis. Journal of Hepatology 2010;52:313–314 10.1016/j.jhep.2009.11.020 20133004

[42] Nast CC, Lemley KV, Hodgin JB, Bagnasco S, Avila-Casado C, Hewitt SM, et al Morphology in the digital age: integrating high-resolution description of structural alterations with phenotypes and genotypes. Seminars in Nephrology 2015; 35:266–278 10.1016/j.semnephrol.2015.04.006 26215864 PMC4764351

