# Supplementary figures and images for: Quantification and Comparison of Anti-Fibrotic Therapies by Polarized SRM and SHG-Based Morphometry in Rat UUO Model
Source: PLoS One. 2016 Jun 3;11(6):e0156734. doi: 10.1371/journal.pone.0156734 (PMC4892485; doi:10.1371/journal.pone.0156734)

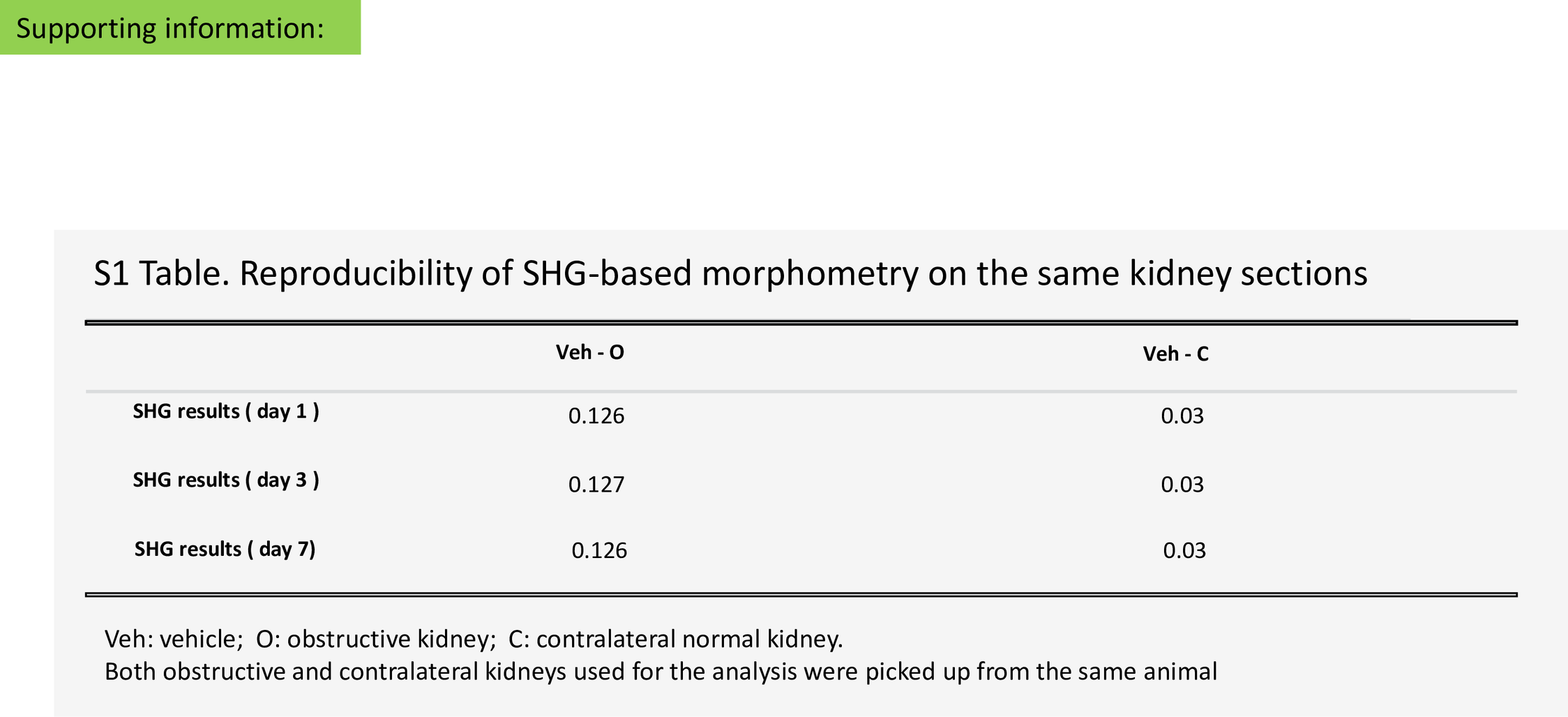

Supplement: S1 Table — The values of cortical IF were calculated by the number of pixels showing intensity above the background threshold relative to the total number of pixels within the ROI. (TIF) [file pone.0156734.s001.tif]
